# Identifying small thymomas from other asymptomatic anterior mediastinal nodules based on CT images using logistic regression

**DOI:** 10.3389/fonc.2025.1590710

**Published:** 2025-07-21

**Authors:** Wenfeng Feng, Runlong Lin, Wenzhe Zhao, Haifeng Cai, Jingwu Li, Yongliang Liu, Lixiu Cao

**Affiliations:** ^1^ Department of Medical Imaging, The Second Hospital of Hebei Medical University, Shijiazhuang, Hebei, China; ^2^ Department of Nuclear Medicine, The Second Hospital of Dalian Medical University, Dalian, Liaoning, China; ^3^ Department of Central Laboratory, Hebei Key Laboratory of Molecular Oncology, Tangshan, Hebei, China; ^4^ Department of Breast Surgery, Tangshan People’s Hospital, Tangshan, Hebei, China; ^5^ Department of Neurosurgery, Tangshan People’s Hospital, Tangshan, Hebei, China; ^6^ Department of Nuclear Medicine Imaging, Tangshan People’s Hospital, Tangshan, Hebei, China

**Keywords:** thymomas, asymptomatic small anterior mediastinal nodules, CT, multivariate logistic regression, unnecessary thymectomy

## Abstract

**Purpose:**

To develop and validate a logistic regression (LR) model to improve the diagnostic performance of chest CT in distinguishing small (≤3 cm in long diameter on CT) thymomas from other asymptomatic small anterior mediastinal nodules (SAMNs).

**Materials and methods:**

A total of 231 patients (94 thymomas and 137 other SAMNs) with surgically resected asymptomatic SAMNs underwenting plain CT and biphasic enhanced CT from January 2013 to December 2023 were included and randomly allocated into training and internal testing sets at a 7:3 ratio. Clinical and CT features were analyzed, and a predictive model was developed based on independent risk features for small thymomas using multivariate LR in the training set. Receiver operating characteristic (ROC) curves and decision curve analysis (DCA) were used to compare the performance of the model and individual risk factors in the internal testing set. An additional prospective testing set (10 thymomas and 13 other SAMNs) was collected from the same institution between 2023 and 2024. The model’s performance was evaluated by area under the curve (AUC) and compared with the results of three radiologists using the DeLong test.

**Results:**

The LR model incorporating four CT independent risk features (lesion location, attenuation pattern, CT values in the venous phase [CTV], and enhancement degree) achieved an AUC of 0.887 for small thymomas prediction. This performance was superior to CTV alone (AUC = 0.849, P = 0.118) and significantly higher than other individual risk factors in the internal testing set (P < 0.05). DCA confirmed the model’s enhanced clinical utility across most threshold probabilities. In the prospective test set, the LR showed an AUC of 0.908 (95% CI: 0.765-1.00), comparable to the senior radiologist’s performance (AUC = 0.912 [95% CI: 0.765-1.00], P = 0.961), higher than the intermediate radiologist’s performance (AUC = 0.762 [95% CI: 0.554-0.969], P = 0.094), and significantly better than the junior radiologist’s performance (AUC = 0.700 [95% CI: 0.463-0.937], P = 0.044).

**Conclusions:**

The CT-based LR model demonstrated well diagnostic performance comparable to that of senior radiologists in differentiating small thymomas from other asymptomatic SAMNs. CTV played a leading role in the model.

## 1 Introduction

With the increasing use of conventional chest CT, there has been a trend toward higher detection rates of asymptomatic anterior mediastinal lesions (1–4). As the most common anterior mediastinal tumor, all thymomas, including low-risk subtypes (A, AB, and B1), and high-risk subtypes (B2 and B3), are currently considered malignant neoplasms (5). Therefore, active surgical intervention remains warranted for thymomas due to their unpredictable clinical behavior, even in asymptomatic patients. While non-therapeutic thymectomy rates reached 22-44% in previous studies (6, 7), this may primarily stem from imaging mischaracterization of other anterior mediastinal pathologies (e.g., thymic cysts, thymic hyperplasia, and lymphoma) as thymomas. Consequently, accurate preoperative differentiation of thymomas using traditional imaging modalities is essential for guiding personalized therapeutic approaches.

CT has been conventionally used for the primary evaluation of anterior mediastinal lesions. However, distinguishing small thymomas (long diameter [LD] ≤3cm) from other anterior mediastinal lesions remains challenging due to overlapping imaging features. Wang et al. demonstrated that certain biologically benign thymic cysts with a diameter ≤3cm can exhibit CT features similar to thymomas (8). The possibility of misdiagnosis mainly stems from two imaging related factors: Firstly, small thymic cysts located near the aorta and sternum may show pseudo enhancement on enhanced CT, leading to the erroneous appearance of solid enhancement features of thymomas; Secondly, based solely on attenuation values, protein rich thymic cysts presenting with solid density on CT may be difficult to distinguish from low enhanced (hypovascular) thymomas. Notably, most incidentally detected anterior mediastinal lesions fall within this small size category (1, 2, 4). Magnetic resonance imaging (MRI) provides higher soft-tissue contrast resolution compared to CT, but its longer acquisition time increases susceptibility to motion artifacts by patient non-cooperation, thereby affecting image quality. In addition, the inherent lower spatial resolution of MRI compared to CT reduces its diagnostic accuracy in characterizing small anterior mediastinal nodules (SAMNs) (9–11). Finally, MRI is more expensive than CT and imposes a greater burden on patients. Although positron emission tomography (PET)/CT is becoming increasingly important in the diagnosis of mediastinal tumors, its clinical application is still limited due to the high cost and limited availability in many centers (12). Furthermore, biopsy procedures carry inherent risks given the proximity of anterior mediastinal lesions to vital cardiovascular structures (13, 14). Given these limitations, there is an urgent need to develop a non-invasive, cost-effective, and reliable method for predicting thymomas in patients with asymptomatic SAMNs.

The current consensus established by the National Comprehensive Cancer Network guidelines (15) states contrast-enhanced CT (CECT) is the gold standard imaging modality for mediastinal tumor evaluation, as it achieves the best balance between reliability, cost-effectiveness, and diagnostic accuracy. However, to our knowledge, only a limited number of studies (16–18) have investigated the CECT’s diagnostic performance for SAMNs. In addition, these studies were limited by sample selection bias, small sample sizes, or lack of comparison with radiologists’ interpretations. Critically, there is a paucity of studies on using machine learning algorithms based on CT to develop a model that can distinguish small thymomas from other SAMNs (including thymic cysts, thymic hyperplasia, and lymphoma) in a large number of patient cohorts with histopathological confirmation.

Therefore, the purpose of this study was to develop a predictive model based on conventional CT features using multivariate LR to effectively identify small thymomas in asymptomatic SAMNs, thereby reducing unnecessary thymectomy and alleviating the related economic and psychological burden on patients.

## 2 Materials and methods

### 2.1 Patients

**Figure 1:**
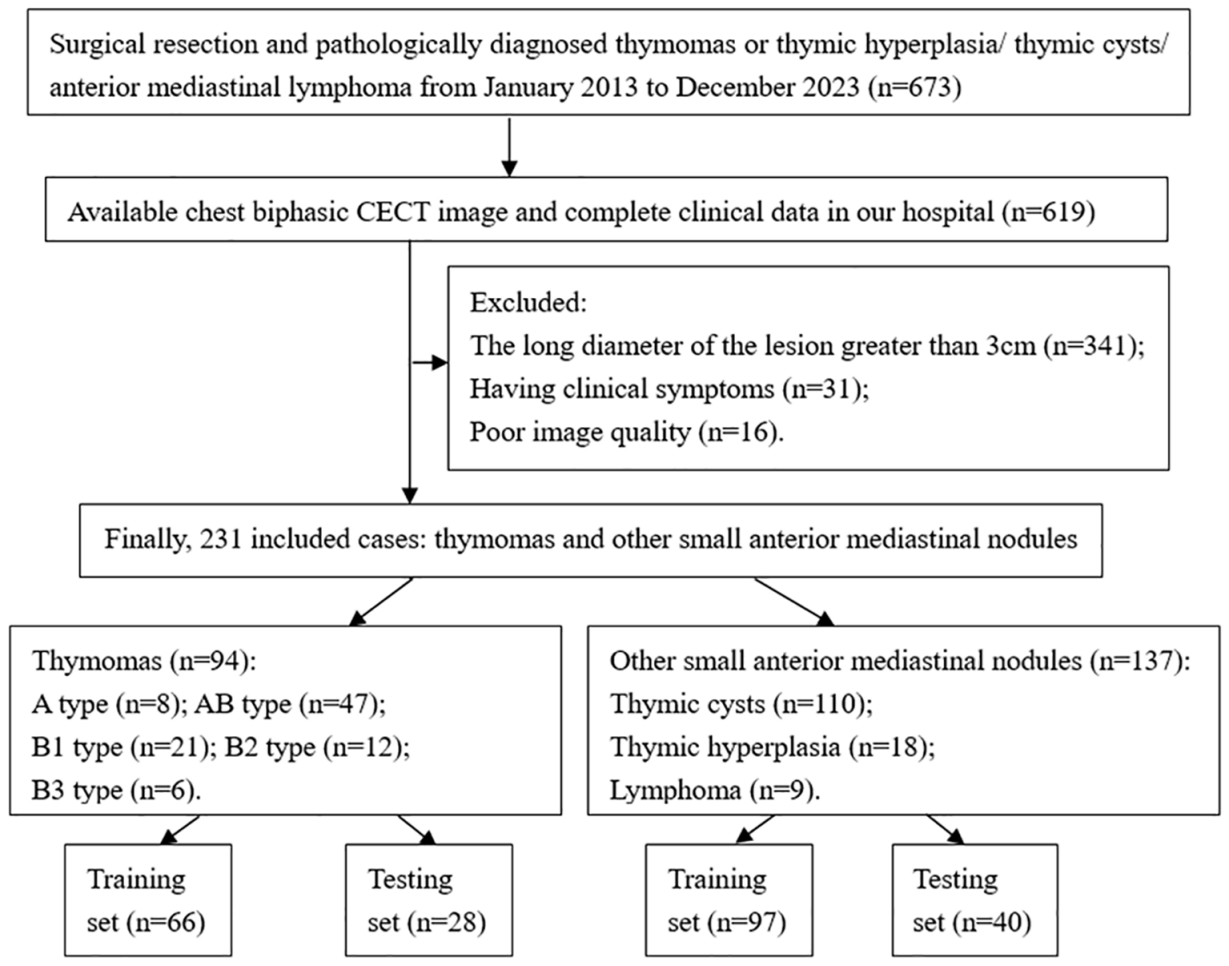
The flow diagram of sample selecting.

This retrospective study was approved by the Institutional Ethics Committee of Tangshan People’s Hospital (Approval No. RMYY-LLKS-2024076) and conducted in accordance with the Declaration of Helsinki (2013 revision). Written informed consent was waived for retrospective data collection but obtained from all prospectively enrolled participants. For the retrospective cohort (January 2013-December 2023), inclusion criteria were: (1) Histologically confirmed diagnosis of small thymoma (LD ≤3cm) or other SAMNs (LD ≤3cm), including thymic cysts, thymic hyperplasia, and lymphoma; (2) Preoperative plain CT and biphasic CECT performed within 14 days prior to surgery, with no history of prior therapy; (3) Asymptomatic clinical presentation; (4) Complete clinical and imaging records; (5) Diagnostic quality CT images (defined as images without significant motion artifacts and with appropriate contrast opacification). The final retrospective cohort included 231 patients (94 thymomas; 137 other SAMNs) who were randomly assigned to a training (n=163; 66 thymomas, 97 other SAMNs) and an internal validation (n=68; 28 thymomas, 40 other SAMNs) sets using a 7:3 allocation ratio (**Figure 1**). Demographic characteristics (age and sex) were recorded and compared between groups.

The prospective validation cohort (January-December 2024) enrolled patients who met the following criteria: a) Asymptomatic SAMNs were incidentally detected on plain CT and biphasic CECT; b) Underwent surgical resection without prior biopsy or treatment (including intervention or targeted therapy). Finally, 23 patients were prospectively enrolled (median age: 54 ± 15 years; 8 males). Pathological diagnoses included small thymomas (n=10: type A=1, AB=6, B1 = 1, B2 = 2) and other SAMNs (n=13: thymic cysts=11, thymic hyperplasia=2).

### 2.2 Image protocol

CT examinations were performed using three scanner models: GE Discovery CT750 HD (GE Healthcare, Milwaukee, WI, USA), Philips Ingenuity Core 16, and Ingenuity Core 64 (Philips Healthcare, Best, Netherlands). The imaging protocol consisted of plain CT scan and biphasic CECT scan, including arterial phase (23 ± 2 seconds) and venous phase (56 ± 4 seconds). A total of 100 mL of non-ionic contrast agent (Iopamidol, 370 mg I/mL) was injected intravenously at 3 mL/s using a dual chamber power injector, followed by 20 mL saline flush. Detailed scanning parameters, including tube voltage, current, and reconstruction settings, are summarized in **Table 1**.

**Table 1:** The scanning parameters and image reconstruction for chest CT scan.

Scanning parameters	GE 64-MDCT chest biphasic CECT scan	Philips 16 and 64-MDCT chest biphasic CECT scan
Tube current	Automated tube current	Automated tube current
Tube voltage	120kV	120kV
Reconstruction algorithm	Soft tissue standard algorithm	Soft tissue standard algorithm
Slice thickness	5mm	2mm
Slice increment	5mm	2mm
Reconstructed thickness	1.25mm	
Reconstructed thickness	1.25mm	
Reconstruction Matrix	512×512	512×512
Number of cases	157	97

### 2.3 Imaging analysis

Two board certified radiologists with 6 and 10 years of thoracic imaging experience independently evaluated the CT features without knowledge of pathological diagnosis and clinical information. The assessment included: (1) Morphology: classified as regular (round/oval) or irregular (all other shapes); (2) Margin: categorized as well-defined or ill-defined; (3) Location: midline (lesion center directly posterior to sternum) or off-midline; (4) Size: LD and short diameter (SD); (5) Attenuation pattern: homogeneous or heterogeneous; (6) Presence of calcifications; (7) Enhancement characteristics: CT values in the unenhanced phase (CTU), arterial phase (CTA), and venous phase (CTV); Presence of cystic/necrotic components (non-enhancing low-attenuation areas). For quantitative analysis, circular regions of interest (ROIs) encompassing approximately 60-70% of the largest cross-sectional area were placed, carefully avoiding artifacts, calcifications, cystic/necrotic areas, and lesion peripheries. Three independent measurements were obtained and the average value was recorded as the final result. Enhancement degree was classified as follows: mild to moderate: enhancement increase <25 HU, moderate to severe: enhancement increase ≥25 HU, respectively (19). The peak enhancement phase was determined based on the maximum enhancement difference between arterial and venous phases: equal enhancement: interphase difference <5 HU; otherwise, the phase in which the maximum enhancement level was defined as the peak enhancement phase (20). Disagreements between the two radiologists were settled by consensus when evaluating CT features.

### 2.4 Observer study

Three radiologists independently evaluated the probability of thymomas in asymptomatic SAMNs within the prospective validation cohort. The evaluation panel comprised one senior radiologist (11 years of thoracic imaging experience), one intermediate radiologist (8 years of experience), and one junior radiologist (2 years of experience). Before evaluation, a thoracic imaging professor with more than 4000 cases of anterior mediastinal lesions review experience provided a training course for the three radiologists. This included a detailed analysis of 20 representative cases from our internal testing set, covering key imaging features and diagnostic criteria. To ensure unbiased assessment, all CT images were anonymous and presented in random order, and radiologists are unaware of all clinical and pathological information. Each clinician provided a probability estimate of thymoma ranging from 0% to 100%.

### 2.5 Statistical analysis and model development

All statistical analyses were performed using R software (version 4.4.2; R Foundation for Statistical Computing). Interobserver consistency was assessed using kappa for categorical features and intraclass correlation coefficients (ICCs) for continuous variables. The consistency level were interpreted as follows: Kappa values or ICCs of 0-0.20 as poor agreement, values of 0.21-0.40 as fair consistency, values of 0.41 and 0.60 as moderate consistency, values of 0.61 and 0.80 as good consistency, values greater than 0.81 as excellent consistency. Continuous variables were compared using Mann-Whitney U test, while categorical variables were analyzed using Fisher exact test or χ² test. Variables with p < 0.1 in univariable analyses underwent multivariable LR with backward elimination, and then the identified independent risk factors for thymomas were used to develop a LR model in the training cohort. Model validation was subsequently performed on both the internal validation set and prospective cohort. Model performance was assessed using receiver operating characteristic (ROC) curve analysis, and clinical utility was evaluated using decision curve analysis (DCA). The DeLong test was used to compare areas under the ROC curves (AUCs) between the model and clinician assessments. A two-tailed P value <0.05 was considered statistically significant.

## 3 Results

### 3.1 Interobserver consistency

The kappa values for categorical CT variables between the two radiologists were as follows: morphology 0.885, margin 0.848, location 1.000, attenuation pattern 0.874, calcification 0.944, cystic/necrotic 0.879, enhancement degree 0.902, peak enhanced phase 0.897. The ICCs for continuous CT variables between the two radiologists were as follows: LD 0.964, SD 0.936, CTU 0.948, CTA 0.970, CTV 0.968.

### 3.2 Comparison of clinical characteristics and CT imaging features in the retrospective sets

There were no significant differences in clinical or CT imaging variables between the training and internal testing sets, confirming the validity of random data grouping (**Table 2**; all p* > 0.05). There were statistically significant differences between small thymomas and other SAMNs in terms of lesion location, morphology, attenuation pattern, three-phase CT values, enhancement degree, cystic/necrotic, and peak enhancement phase (all p<0.05); However, no significant differences were observed in patient age, gender, lesion margin, SD, LD, and calcification (all p>0.05) (**Table 2**). Specifically, in the training and testing sets, 78.79% and 78.57% of thymomas were located off-midline, while only 50.52% and 52.50% of other SAMNs were located off-midline. The proportion of irregular shapes in thymomas was significantly higher in both the training (57.58% vs. 28.87%, p<0.001) and testing (64.29% vs. 40.00%, p=0.049) sets compared to other SAMNs. More than half of thymomas showed heterogeneous on plain CT scan, while most other SAMNs were homogeneous in both the training and testing sets. **T**he CTU, CTA, and CTV values of thymomas were significantly higher than those of other SAMNs. In addition, 77.27% and 75.00% of thymomas showed moderate to severe enhancement in the training and testing sets, respectively, while other SAMNs were only 18.56% and 20.00%. The cystic/necrotic rate of thymomas was also significantly higher than that of other SAMNs. The peak enhancement of thymoma mainly occurred in the venous phase (68.18% of training and 78.57% of testing), while other SAMNs mainly occurred in the venous phase (42.27% of training and 50.00% of training) and equal enhancement (43.30% of training and 43.30% of training).

**Table 2:** Clinical and CT features of the patients in the retrospective sets.

Features	Training set (n=163)			Internal testing set (n=68)			P*value
	Thymomas (n=66)	Other SAMNs (n=97)	*P* value	Thymomas (n=28)	Other SAMNs (n=40)	*P* value	
Age(years)	54.1 [46,64]	51.6 [43,59]	0.052	55.1[48.5,61.8]	51.6[43.8,57.2]	0.051	0.932
Sex			0.620			0.649	0.363
Female	40 (60.61%)	55 (56.70%)		19 (67.86%)	25 (62.50%)		
Male	26 (39.39^)	42 (43.30%)		9 (32.14%)	15 (37.50%)		
Morphology			<0.001			0.049	0.184
Regular	28 (42.42%)	69 (71.13%)		10 (35.71%)	24 (60.00%)		
Irregular	38 (57.58%)	28 (28.87%)		18 (64.29%)	16 (40.00%)		
Margin			0.188			>0.999	0.631
Well-defined	48 (72.73%)	79 (81.44%)		21 (75.00%)	30 (75.00%)		
Ill-defined	18 (27.27%)	18 (18.56%)		7 (25.00%)	10 (25.00%)		
Location			<0.001			0.028	0.856
Midline	14 (21.21%)	48 (49.48%)		6 (21.43%)	19 (47.50%)		
Off-midline	52 (78.79%)	49 (50.52%)		22 (78.57%)	21 (52.50%)		
LD	2.39[2,2.88]	2.36[1.8,2.9]	0.964	2.38[1.98,2.9]	2.36[1.9,2.8]	0.586	0.788
SD	1.96[1.5,2.4]	1.94[1.4,2.5]	0.904	1.92[1.5,2.3]	1.93[1.6,2.4]	0.906	0.748
Attenuation pattern			<0.001			0.002	0.617
Homogeneous	30 (45.45%)	81 (83.51%)		12 (42.86%)	32 (80.00%)		
Heterogeneous	36 (54.55%)	16 (16.49%)		16 (57.14%)	8 (20.00%)		
Calcification			0.081			0.161	0.215
Yes	15 (22.73%)	12 (12.37%)		9 (32.14%)	7 (17.50%)		
No	51 (77.27%)	85 (87.63%)		19 (67.86%)	33 (82.50%)		
CTU	42.7[36.8,49]	30.6[19,42]	<0.001	39.8[33.8,45]	30.8[19.8,40.5]	0.007	0.486
CTA	72.4[59,86.5]	43.6[30,56]	<0.001	66.8[54.8,81.5]	39.5[27.8,49.2]	<0.001	0.193
CTV	82.1[70,93]	46[33,58]	<0.001	76.6[67,93.2]	46.2[30.5,57.2]	<0.001	0.580
Cystic/necrotic			<0.001			<0.001	0.935
Yes	39 (59.09%)	20 (20.62%)		17 (60.71%)	8 (20.00%)		
No	27 (40.91%)	77 (79.38%)		11 (39.29%)	32 (80.00%)		
Enhancement degree			<0.001			<0.001	0.965
Mild-moderate	15 (22.73%)	79 (81.44%)		7 (25.00%)	32 (80.00%)		
Moderate-severe	51 (77.27%)	18 (18.56%)		21 (75.00%)	8 (20.00%)		
Peak enhanced phase			0.004			0.021	0.231
Arterial phase	7 (10.61%)	14 (14.43%)		2 (7.14%)	2 (5.00%)		
Venous phase	45 (68.18%)	41 (42.27%)		22 (78.57%)	20 (50.00%)		
Equally enhanced	14 (21.21%)	42 (43.30%)		4 (14.29%)	18 (45.00%)		

*p* value < 0.05 indicates a significant difference between thymomas and other SAMNs in the training or internal testing set.

p* value < 0.05 indicates a significant difference between the training and internal testing sets.

SAMNs Small anterior mediastinal nodules, LD Long diameter, SD Short diameter, CTU CT values in the unenhanced phase, CTA CT values in the arterial phase, CTV CT values in the venous phase.

### 3.3 Predictive model

**Figure 2:**
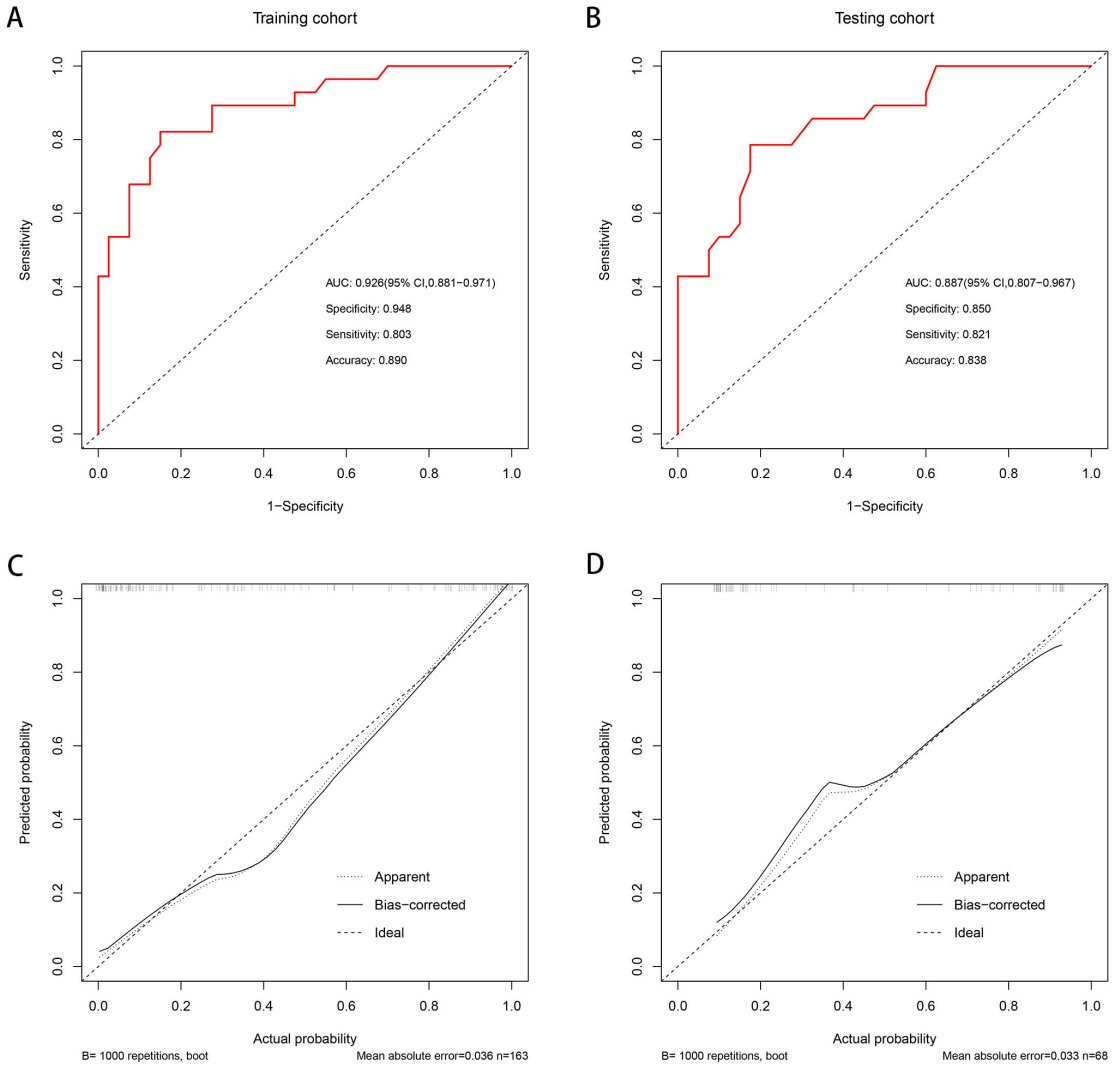
The AUC of the predictive model was 0.926 [95% CI 0.881-0.971], with a sensitivity, specificity and accuracy of 0.803, 0.948, and 0.890 in the training set, respectively **(A)**. The AUC of the predictive model was 0.887 [95% CI 0.807-0.967], with a sensitivity, specificity, and accuracy of 0.821, 0.850, and 0.838 in the internal testing set, respectively **(B)**. Good calibrations of the predictive model were shown in both the training **(C)** and internal testing sets **(D)**.

**Figure 3:**
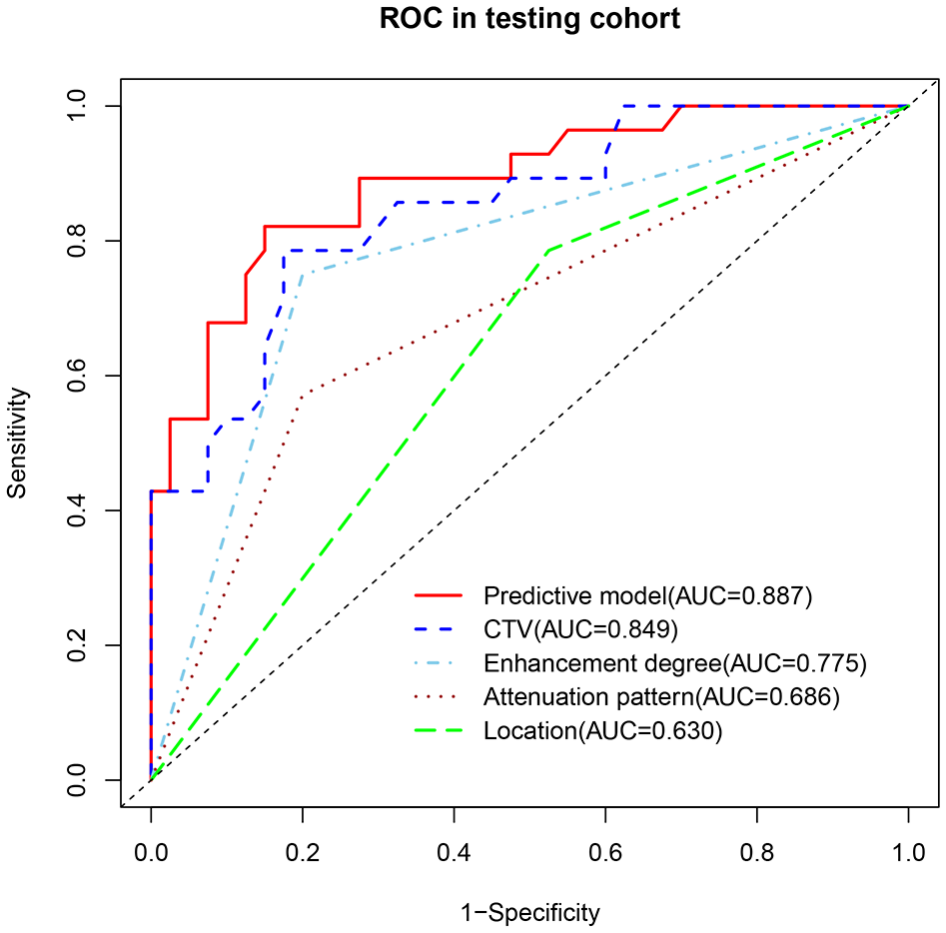
The AUC of the predictive model was higher than that of any individual risk factor in the internal testing set.

**Figure 4:**
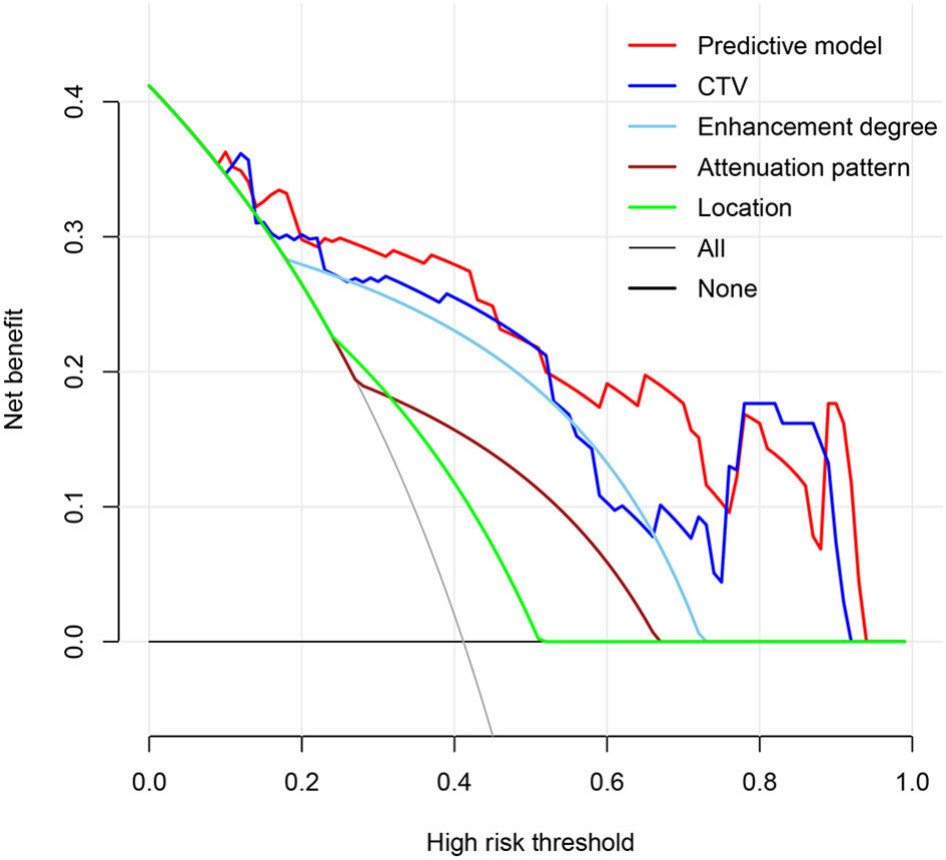
The predictive model exhibited enhanced clinical utility across most threshold probabilities when compared with independent risk factors in the internal testing set.

**Figure 5:**
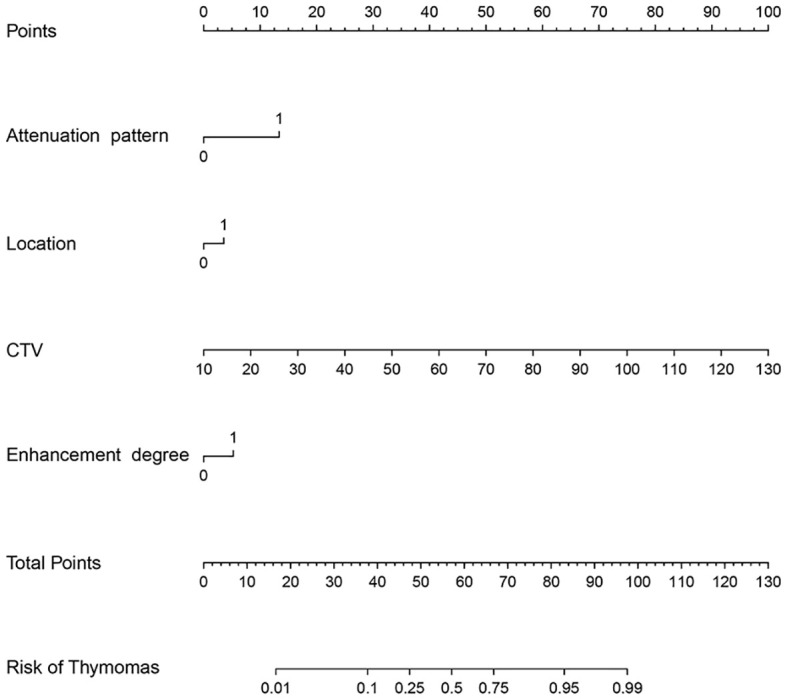
Nomogram of the predictive model.

To avoid redundancy due to strong collinearity among CTA, CTU, and CTV (**Supplementary Figure 1**), only CTV was retained for subsequent analysis because of the highest AUC value. Nine variables with p < 0.1 at univariable analyses (including age, morphology, location, attenuation pattern, calcification, CTV value, cystic/necrotic, enhancement degree, and peak enhancement phase) underwent multivariable LR with backward elimination. Four independent risk factors of small thymomas (including location, attenuation pattern, CTV value, and enhancement degree) were used to develop the final LR model. In the training set, the AUC of the LR model was 0.926 [95% confidence interval (CI) 0.881-0.971], with specificity, sensitivity, and accuracy of 0.948, 0.803, and 0.890, respectively. In the testing set, its AUC was 0.887 (95% CI 0.807-0.967), with specificity, sensitivity, and accuracy of 0.850, 0.821, and 0.838, respectively (**Figures 2A, B**). Calibration curves demonstrated good agreement between predicted and observed probabilities in both cohorts (**Figures 2C, D**). Although the AUC of the LR model was higher than that of CTV, there was no significant difference (0.887 vs. 0.849, P=0.118); However, it was significantly higher than any other risk factor (P < 0.05; **Table 3**, **Figure 3**). DCA further indicated that compared to independent risk factors in the same cohort, the LR model exhibited stronger clinical utility across most threshold probabilities (**Figure 4**). The related nomogram showed that a lesion exceeding 58.15 could be considered a small thymoma, with an AUC of 0.926, specificity of 94.8%, sensitivity of 80.3%, and overall accuracy of 89.0% (**Figure 5**).

**Table 3:** The comparison of the AUCs between the predictive model and individual risk factors in the internal testing set.

Comparison	AUC	Z statistic	*P* value
Predictive model vs CTV	0.887 vs 0.849	1.563	0.118
Predictive model vs Enhancement degree	0.887 vs 0.775	2.953	0.003
Predictive model vs Attenuation pattern	0.887 vs 0.686	3.619	<0.001
Predictive model vs Location	0.887 vs 0.630	4.026	<0.001

AUC area under the receiver operating characteristic curve, CTU the value on unenhanced CT.

### 3.4 Comparison of the diagnostic performance between the nomogram and radiologists in the prospective testing set

**Figure 6:**
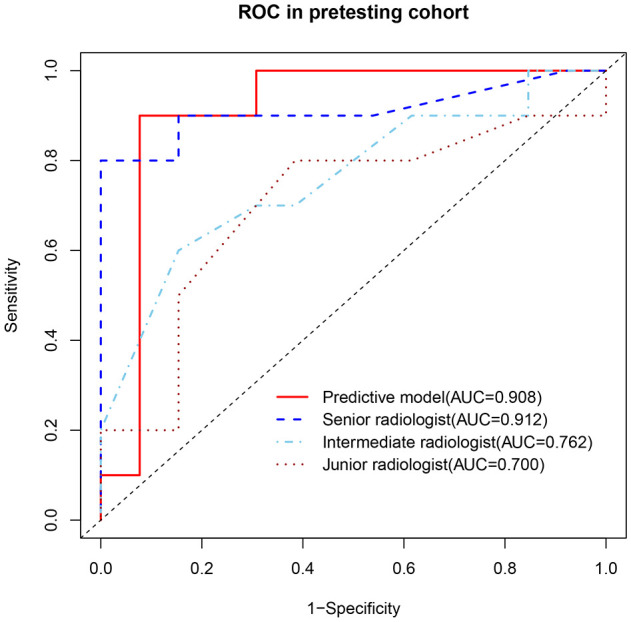
ROCs of the predictive model and radiologists in identification of thymomas from other small (≤3 cm) anterior mediastinal nodules in the prospective testing set.

**Figure 7:**
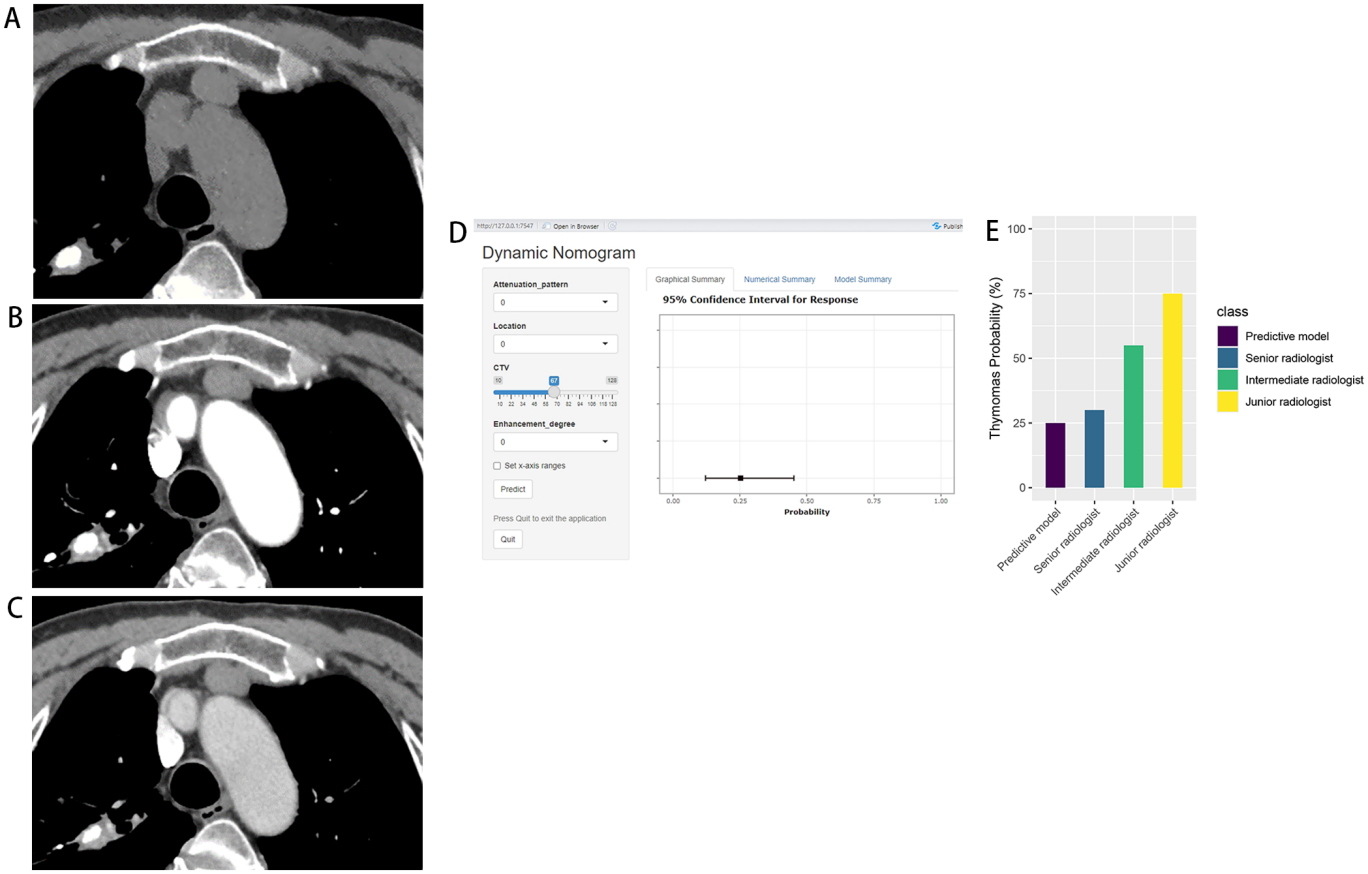
Thymic cyst in a 65-year-old woman. Axial CT scans in unenhanced **(A)**, arterial **(B)**, and venous **(C)** phases demonstrate a 2.0cm×1.8cm homogeneous anterior mediastinal nodule with middle location. The CTU, CTA, and CTV values of the lesion were 43HU, 55HU, and 67HU, respectively. Application of this case to the diagnostic nomogram **(D)** yielded a score of 47.5 points, corresponding to a 25% probability of thymoma. Graph **(E)** depicts that the predictive model exhibited higher confidence in diagnosing the lesion as non-thymoma compared with the senior radiologist (30%), whereas the intermediate radiologist (55%) and the junior radiologist (75%) were more inclined toward thymoma.

In the prospective testing set, the nomogram achieved an AUC of 0.908 (95% CI: 0.765-1.00). At 90% specificity, the sensitivity was 90% (9 of 10 thymomas; 95% CI: 0, 100). The nomogram’s performance was comparable to that of the senior radiologist for both AUC (0.908 vs. 0.912, P = 0.961) and sensitivity at 90% specificity (90% vs. 90%, P > 0.999). Compared with the intermediate radiologist, the nomogram showed higher AUC (0.908 vs. 0.762, P = 0.094) and sensitivity at 90% specificity (90% vs. 50%, P = 0.141). In addition, compared with the junior radiologist, the nomogram demonstrated significantly higher AUC (0.908 vs. 0.700, P = 0.044) and sensitivity at 90% specificity (90% vs. 30%, P = 0.020) (**Figure 6**, **Table 4**). One representative case predicted by the nomogram and radiologists are shown in **Figure 7**.

**Table 4:** Performance of the predictive model and radiologists in identification of thymomas in the prospective testing set.

Variable	AUC*	*P* Value	Sensitivity (%)^+^	*P* Value
Predictive model	0.908(0.765,1.000)		90 (9/10)[0, 100]	
Senior radiologist	0.912(0.765,1.000)	0.961	90 (9/10)[60, 100]	> 0.999
Intermediate radiologist	0.762(0.554,0.969)	0.094	50 (5/10)[14, 90]	0.062
Junior radiologist	0.700(0.463,0.937)	0.044	30 (3/10)[0, 82]	0.020

Note. P values correspond to comparisons between the predictive model and each radiologist. Sensitivity values were computed at a specificity of 90%. AUC = area under the receiver operating characteristic curve.

*Data in parentheses are 95% CIs.

+Data in parentheses are numbers of patients; data in brackets are 95% CIs.

## 4 Discussion

In our study, we developed and validated an LR model based on traditional CT imaging features (including attenuation pattern, location, CTV, and enhancement degree) to identify small thymomas in patients with asymptomatic SAMNs. In the prospective testing set, LR model demonstrated robust performance in predicting small thymomas, with its ability comparable to that of the senior radiologist, higher than that of the intermediate radiologist, and significantly improved compared to the junior radiologist. These results suggest that the model’s high diagnostic accuracy could serve as a decision-support tool to reduce unnecessary examinations and non-therapeutic surgeries.

In recent years, few studies have systematically evaluated the diagnostic efficacy of conventional CT features for differentiating anterior mediastinal lesions ≤3 cm in diameter. Jin et al. (16) demonstrated that CECT showed promising diagnostic capability for distinguishing high-density thymic cysts from thymomas in sub-3 cm lesions, with AUC values of 0.95 for enhanced CT values and 0.96 for ΔCT values. But this study was limited by a small cohort (n=36). In addition, no nomogram was developed for providing a quantitative tool to predict an individual’s probability of thymomas. Although Jung et al. (17) developed a CT-based nomogram achieving 95% accuracy in differentiating thymomas from cysts in 100 patients with asymptomatic SAMNs, their study did not compare the nomogram with the diagnosis of radiologists and lacked sufficient statistical power due to moderate sample size. In addition, the two studies mentioned above also had a common limitation: they only distinguished between thymomas and thymic cysts, excluding thymic hyperplasia and lymphoma that need to be differentiated in normal diagnosis. To our knowledge, our study represents the largest sample size to date, incorporating 231 cases from retrospective analysis and 23 prospectively validated cases. Distinctively, our research framework included differential diagnosis between thymomas and various mediastinal pathologies, including thymic cysts, thymic hyperplasia, and lymphomas. And CT data obtained from different scanning machines and scanning parameters. In addition, we implemented a rigorous comparative analysis of the diagnostic performance between the LR model and radiologists with different experience levels. These methodological advances may potentially enhance the model’s generalizability and clinical robustness for distinguishing thymomas from other asymptomatic SAMNs.

CTV and enhancement degree were two independent predictive factors in our LR model for differentiating small thymomas from thymic cysts, hyperplasia, and lymphomas. Both demonstrated statistically significant discriminative ability. Thymomas exhibited significantly higher CTV values compared to other SAMNs, consistent with previous study (21). For instance, Xie et al. (21) observed that higher iodine concentrations in enhancing thymomas versus mediastinal lymphomas during the venous phase. Furthermore, thymomas were predominantly characterized by moderate to severe enhancement, while other SAMNs typically showed mild to moderate enhancement. This aligned with established findings (5, 16, 22, 23). Jung et al. (17) specifically identified enhancement degree as a key factor in distinguishing thymomas and cysts in small (<3 cm) thymic lesions. Priola et al. (24) also reported that enhancement degree was a risk factor in distinguishing thymoma from thymic hyperplasia, and pointed out that thymomas tended to have moderate to severe enhancement. These findings indicated that superior vascularity in small thymomas relative to other SAMNs, likely peaking during the venous phase. This hemodynamic pattern is associated with characteristic histopathology: thymomas are composed of neoplastic epithelial cells of thymus intermingled with non-neoplastic immature T lymphocytes, which are separated into lobules by fibrous bands and contain prominent perivascular spaces (25). These fibrous septa decreases the speed of withdrawal of contrast agents, resulting in a higher iodine concentration during the venous phase. In our analysis, the location of the lesion was also an independent risk factor for small thymomas. Up to 80% of thymomas showed an off-midline position, while only 50% of other SAMNs. This spatial distribution pattern was consistent with existing studies. Ackman et al. (6) reported that 82% of thymomas were off-midline, while 44% for thymic cysts, 26% of lymphoma, and 10% of thymic hyperplasia. Nam et al. (26) also reported that thymomas tended to preferentially occupy an off-midline position, while McErlean et al. (27) concluded that midline position was more common in benign thymic lesions. Attenuation pattern was another key independent predictor in our model. More than 50% of thymomas demonstrated heterogeneity on plain CT, while most other SAMNs showed uniformity, which was consistent with previous studies (7, 28, 29). Araki et al. (28) reported that an inhomogeneous attenuation on plain CT suggested thymoma rather than thymic cyst.

Although the independent risk features mentioned above had a certain discriminatory diagnostic ability when used alone, integrating these findings into a predictive model achieved excellent discriminative ability, comparable to the subjective evaluation of senior radiologists in a prospective cohort. It was worth noting that the diagnostic accuracy of intermediate and junior radiologists in the prospective cohort was limited when relying solely on diagnostic experience. This finding highlights the special diagnostic challenges posed by asymptomatic SAMNs for less experienced radiologists. Furthermore, the inherent variability of diagnostic experience limits the reproducibility when radiologists predict thymomas alone. In contrast, our nomogram-based approach provides an exact probability of thymoma, regardless of the radiologist’s experience. Therefore, even clinical physicians can objectively predict the accurate probability of small thymoma by inputting four easily obtainable CT features (CTV value, enhancement degree, location, and attenuation pattern) into the nomogram. This standardized quantitative evaluation may be more reliable in distinguishing small thymomas from other SAMNs than individual experiential radiological interpretation.

Critically, our LR model facilitates accurate identification of small thymomas among asymptomatic SAMNs, which may reduce unnecessary thymectomy rates. This could generate significant health economic benefits: despite the low mortality rate of non-therapeutic thymectomy, the documented complications and healthcare costs remain a concern. Kent et al. (7) reported that based on a ten-year national sample of hospitalized patients, the total cost of non-therapeutic thymectomy exceeded $16 million. Furthermore, even if surgical recovery is not complicated, indirect costs caused by productivity loss need to be considered.

Nevertheless, this study has several limitations. Firstly, the predictive model was developed using surgical patient data exclusively. Including non-surgical cases could provide a more realistic SAMNs’ distribution, which may enhance the generalizability of the model. Secondly, our study employed a single center design and lacked external validation. Future multicenter research could improve the performance of predictive models to some extent. Thirdly, the small sample size of the prospective test set may lead to a wider confidence interval for model performance estimation and may potentially exaggerate its superiority over radiologists, thereby reducing the reliability of our results. Future research will expand the prospective validation cohort to improve the reliability and clinical applicability of the model. Finally, although the three radiologists received standardized training, potential observer bias remains a limitation. For example, senior radiologist may rely on clinical experience beyond imaging features during the interpretation process. In future research, differences between observers should be formally evaluated at different levels of experience.

In conclusion, our LR model based on conventional CT features can effectively identify small thymomas (≤3cm) in asymptomatic SAMNs, with performance comparable to that of senior radiologists. We believe that this model could facilitate active surveillance for asymptomatic SAMNs and reduce unnecessary thymectomies.

## Data Availability

The raw data supporting the conclusions of this article will be made available by the authors, without undue reservation.
