# Supplementary material for: Identifying small thymomas from other asymptomatic anterior mediastinal nodules based on CT images using logistic regression
Source: Front Oncol. 2025 Jul 21;15:1590710. doi: 10.3389/fonc.2025.1590710 (PMC12318746; doi:10.3389/fonc.2025.1590710)
Supplement: Supplementary file 1 [file DataSheet1.docx]

Supplementary Material

# Supplementary Figures


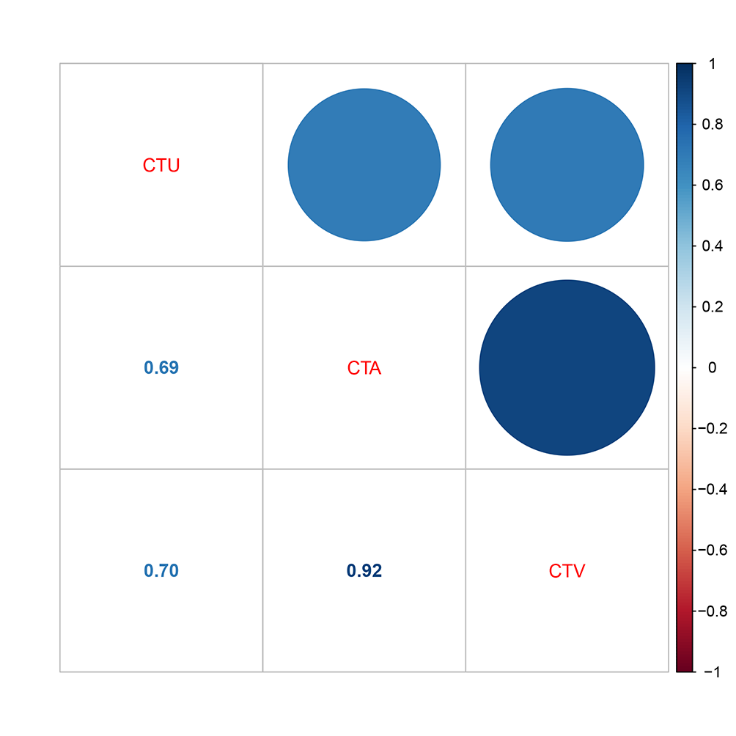


**Supplementary Figure 1.** Strong correlations were observed among CTU, CTA, and CTV.
